# Root system architecture remodeling under oxygen-mediated subsurface drip irrigation: a mechanistic hypothesis framework linking multiphase mass transfer to root foraging decisions

**DOI:** 10.3389/fpls.2026.1951701

**Published:** 2026-09-14

**Authors:** Yijie Zhang, Yongmei Zhao, Yulan Mo, Zhitao Zhang, Wen Ren, Lingqiong Kong

**Affiliations:** 1 College of Mechanical and Electrical Engineering, Yunnan Agricultural University, Kunming, China; 2 College of Water Conservancy, Yunnan Agricultural University, Kunming, China

**Keywords:** aerated subsurface drip irrigation, mechanistic framework, optimal foraging theory, root foraging-accessible domain, root system architecture, soil oxygen transport, subsurface drip irrigation

## Abstract

As climate variability and water scarcity intensify, subsurface drip irrigation (SDI) has emerged as a key water-saving technology across arid and semiarid regions. However, in practice, the wetted soil volume around each emitter remains close to saturation, disrupting the continuity of the soil gas phase and restricting oxygen diffusion into the root zone. Subsequently, the water and oxygen supplies may compete for the same pore space, which is an intrinsic constraint of SDI. Aerated SDI, particularly systems that use micro- and nanobubbles (MNBs), has shown potential to improve crop yield and water-saving performance, although the effects vary with soil, crop, and management conditions. A unified, cross-scale account of the influence of these engineered gas–liquid inputs on root system architecture (RSA) remodeling remains lacking. Therefore, based on the optimal foraging theory (OFT), we propose an oxygen-mediated root foraging cost framework as a working mechanism hypothesis. This framework treats oxygen not as a growth-promoting factor per se, but as a candidate regulatory variable that may modulate the metabolic cost of root resource acquisition; the sensing–signaling–response chain linking local oxygen gradients to architectural decisions remains to be causally validated. By altering foraging costs, oxygen availability may alter how roots perceive, trade off, and invest in heterogeneous underground resources. Guided by this framework, this review first traces how SDI has evolved technologically, from supplying water through the liquid phase to coupling gas and liquid through MNBs. It then examines how local oxygen gradients may be sensed through upstream oxygen-sensing pathways (including the N-end rule pathway), reactive oxygen species (ROS), nitric oxide (NO), and downstream ethylene–auxin interactions, and how these signals may shape patterns of RSA investment under localized SDI conditions, and proposes the root foraging-accessible domain (FAD) as a candidate concept for evaluating the spatial compatibility between root foraging and the coupled water–oxygen environment. The present evidence supports this conceptual framework, but field-scale causal validation, separation of oxygen effects from confounding factors (nutrients, mechanical impedance, microbial activity), and techno-economic evaluation of aerated SDI remain necessary before the framework can guide irrigation practice.

## 1 Introduction

### 1.1 SDI water saving and the root-zone oxygen constraint

Subsurface drip irrigation (SDI) delivers water and nutrients directly to the crop root zone through buried emitters, achieving an irrigation water application efficiency that frequently exceeds 90% (Yao et al., 2021). Compared to conventional methods, it reduces irrigation water use by 35–55% (Lamm and Trooien, 2003), making it a key water-saving technology in arid agricultural systems (Camp, 1998; Lakhiar et al., 2024). A recent global meta-analysis of 109 publications and 984 paired comparisons reported that SDI increased crop yield by 5.39% and irrigation water productivity (IWP) by 6.75% relative to surface drip irrigation (Wang et al., 2022). Beyond yield and water productivity, broader evaluations have also been applied to agricultural water and nutrient management. Under ridge and furrow plastic film mulching on the Loess Plateau, nitrogen management was assessed by considering crop growth, yield, resource use efficiency, economic benefits, and soil quality together (Zhang et al., 2025a). Recently, research attention has shifted from engineering questions, such as emitter hydraulic design and clogging prevention, to the coupled regulation of water, nutrients, and oxygen, along with the management of the rhizosphere microenvironment (Lei et al., 2022; Yao et al., 2021). However, this efficiency results in structural compromise. Around buried emitters, the wetted zones remain close to saturation; therefore, gas-phase connectivity among soil pores declines, and oxygen diffusion into the root zone is notably constrained, particularly in fine-textured soils and under intensive, high-frequency irrigation (Ben-Noah and Friedman, 2018; Bhattarai et al., 2005). This coupling has a well-established physical basis: effective gas-phase diffusivity is a steep function of air-filled porosity (Penman, 1940; Millington and Quirk, 1961), and the relationship is further modulated by pore-size distribution and pore structure and connectivity (Moldrup et al., 2000a; 2000b). Consequently, even moderate increases in volumetric water content can disproportionately reduce oxygen flux to root surfaces. The water supply and oxygen supplies may therefore compete for the same pore space, which is a physical constraint intrinsic to the SDI.

To mitigate this effect, Bhattarai et al. (2006, 2005) introduced the delivery of air-or oxygen-enriched water into the root zone using SDI systems. Aerated SDI has attracted increasing interest over the past two decades, particularly in systems based on Venturi air injection and micro-and nanobubbles (MNBs). Meta-analyses have reported positive agronomic responses, including yield increases of 13.55% and 18.6% (Jiao et al., 2026a; Wang et al., 2026b) and a water-use efficiency gain of 17.9% (Du et al., 2018), but the magnitude of the response varies across soil, crop, and management conditions. Owing to their high interfacial energy, long residence time, and a strong negative surface charge (Chen et al., 2025a), MNBs have progressed from laboratory demonstrations to potential commercial deployment in protected fertigation systems (Zheng et al., 2025a). However, improved dissolved oxygen in irrigation water should not be treated as a direct proxy for oxygen delivery to root surfaces or for a resulting yield benefit. Nonetheless, a mechanistic understanding of MNB transport and retention in heterogeneous field soils, as opposed to controlled laboratory conditions, remains a major challenge (Borsky et al., 2024).

### 1.2 From aerated SDI to oxygen-mediated root foraging

The chain of events linking oxygen transport at the pore scale to root architectural decisions is only partially understood, making it difficult to reliably translate this technology across different soils, crops, and environmental conditions. Previous reviews have investigated aerated drip irrigation from different perspectives, including aeration delivery devices (Li et al., 2025a), the physicochemical behavior of MNBs (Zheng et al., 2025a; Zhang et al., 2023a), and the ways in which emitter placement modifies the spatial distribution of roots (Qian et al., 2025). However, the integration of multiphase flow and mass transfer, rhizosphere oxygen dynamics, and root system architecture (RSA) plasticity into a unified, plant-centered mechanistic framework has not been sufficiently addressed. While irrigation hydraulics and root biology provide established frameworks for understanding their respective mechanisms, the cross-scale causal chain linking irrigation engineering inputs to RSA remodeling and spatial resource allocation remains poorly understood. Yield gains from aerated SDI typically result from an increased supply of dissolved oxygen (DO) in the root zone. However, root responses to hypoxia and resource heterogeneity cannot be attributed solely to elevated oxygen concentrations. At the rhizosphere and root tissue scales, local oxygen gradients can modulate the balance between reactive oxygen species (ROS) production and scavenging, thereby influencing hypoxia signaling and RSA remodeling. These responses may also involve upstream oxygen sensing and downstream redox- and hormone-related signaling, although most supporting evidence comes from systems unrelated to localized SDI conditions (Licausi et al., 2011; Drew, 1997; Karlova et al., 2021; Morris et al., 2017). Therefore, the critical factor is not the absolute oxygen level in the bulk root zone but the spatial coupling among oxygen availability, redox homeostasis, and sites of active root growth (Jiao et al., 2026b). The local moisture signal introduces another dimension; Through hydropatterning and auxin mediated development processes, they affect lateral root localization (Giehl and von Wiren, 2018), suggesting that root systems may respond to the heterogeneity of water and oxygen caused by SDI through two pathways: stress signal transduction and spatial development pattern formation. However, most of this evidence is derived from whole-root-zone treatments or settings unrelated to SDI; therefore, whether the localized hypoxic microdomains around SDI emitters can generate spatially heterogeneous hormone gradients by themselves remains unclear.

The optimal foraging theory (OFT) provides a complementary explanation. The extent to which a root invests in a heterogeneous resource patch depends on the local return, the construction and maintenance costs associated with deploying roots in that patch, and the opportunity cost of alternative patches not exploited (MacArthur and Pianka, 1966; Charnov, 1976). OFT predicts that a forager’s investment in a given resource patch depends on the marginal return, the construction and maintenance costs of exploiting that patch, and the opportunity cost of unexploited alternatives (MacArthur and Pianka, 1966; Charnov, 1976; McNickle et al., 2009). Applied to root systems, this logic implies that roots preferentially proliferate in patches where the net return per unit cost is highest (McNickle et al., 2009), and that localized nutrient enrichment can trigger localized lateral root proliferation (Drew and Saker, 1975). After the marginal returns in a patch decline, growth tends to migrate toward sites that offer increased potential returns (McNickle et al., 2009; McNickle and Cahill, 2009; Renton et al., 2025). Thus, aerated SDI may influence RSA plasticity less by increasing the oxygen concentration than by reducing the metabolic cost of foraging inside the resource-enriched zones. However, nutrient availability, mechanical impedance, soil structure, microbial activity, and temperature also modulate root deployment costs independently of oxygen (Drew and Saker, 1975; Grable and Siemer, 1968); therefore, the oxygen-mediated cost reduction proposed here represents one candidate pathway among several that may operate simultaneously. We therefore treat oxygen-mediated foraging cost as a working hypothesis linking the engineering inputs of aerated SDI to RSA remodeling, rather than as an established causal mechanism.

### 1.3 Literature-development evidence and the unresolved cross-scale gap

To examine how the field has developed, we conducted a bibliometric analysis of research articles and reviews indexed in the Web of Science (WoS) and China National Knowledge Infrastructure (CNKI) from January 2000 to December 2025. Keyword co-occurrence was mapped using VOSviewer 1.6.20 with a minimum occurrence threshold of five. The analysis identifies how research emphasis has shifted from hydraulic engineering toward oxygen regulation and root biology, and locates the disciplinary interfaces where causal evidence remains sparse.

The publication record shows a shift in research emphasis. Early work was centered on soil water movement and hydraulic regulation; subsequent studies increasingly incorporated water–nutrient interactions; and more recent work has focused on oxygen regulation, rhizosphere responses, MNBs, and quantitative evaluation of aerated irrigation (**Figure 1**). This trend confirms that the field has expanded toward oxygen and rhizosphere regulation, yet the causal link between engineering oxygen inputs and root architectural responses remains weakly represented in the literature.

**Figure 1:**
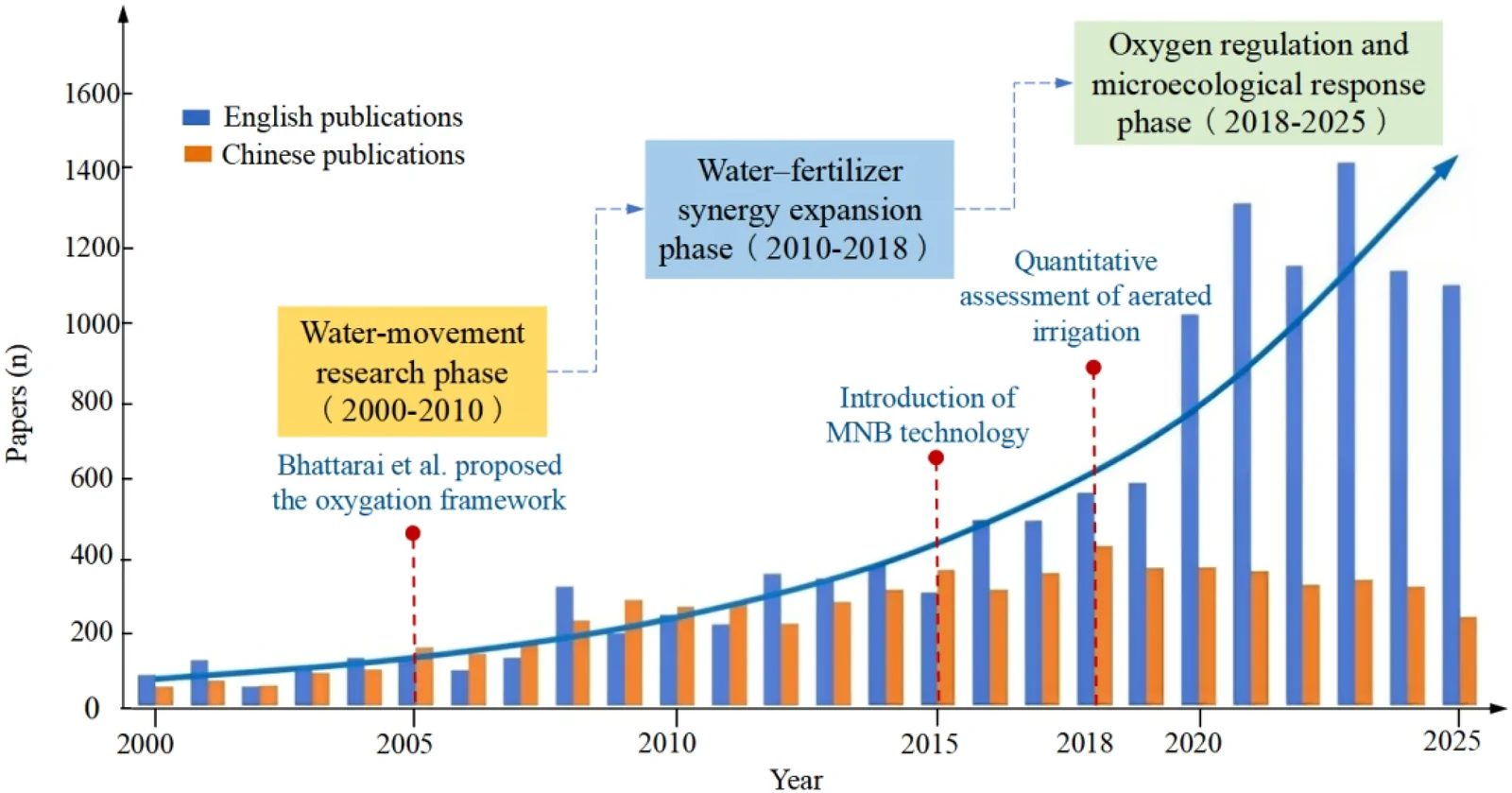
Annual publication trends and the evolution of technological paradigms.

The co-occurrence network shows three features relevant to this review (**Figure 2**). First, MNB and water-treatment topics form a comparatively distinct engineering cluster, whereas RSA and plant-growth topics form a plant-related cluster with only limited direct connections between them. Second, RSA plasticity appears near the interface between hydraulic regulation, MNB-related research, and yield formation, suggesting that it could serve as a biological connector between engineering inputs and crop outcomes. Third, irrigation-system modeling and numerical simulation remain weakly connected to spatially and temporally explicit root physiological measurements. The network does not establish causality; it identifies the disciplinary interface between multiphase flow engineering and root ecophysiology, where mechanistic evidence is most needed. Three points of consensus frame this review: SDI creates a structural trade-off between water delivery and oxygen supply, governed by the steep dependence of gas-phase diffusivity on air-filled porosity (Penman, 1940; Millington and Quirk, 1961; Moldrup et al., 2000b); aerated SDI and MNB systems can raise dissolved oxygen and have been linked to positive agronomic responses; and root systems adjust their architecture to local resource heterogeneity in ways consistent with optimal foraging logic (MacArthur and Pianka, 1966; Charnov, 1976). Three gaps persist. MNB behavior in heterogeneous field soils remains poorly characterized; *in situ* evidence linking emitter-scale oxygen gradients to root sensing-signaling-response pathways is lacking; and oxygen effects on RSA have not been isolated from nutrients, mechanical impedance, and microbial activity. We therefore propose, as a working hypothesis, that oxygen under aerated SDI influences RSA plasticity primarily by reducing the metabolic cost of root foraging rather than by promoting growth directly. This review traces the shift from pressure-driven water delivery to gas-liquid coupling, reassesses RSA responses through OFT cost-benefit logic, and introduces the root foraging-accessible domain (FAD) for evaluating compatibility between root foraging and the water-oxygen environment. Validation requires field-scale experiments that simultaneously resolve oxygen dynamics, architectural responses, and confounding variables.

**Figure 2:**
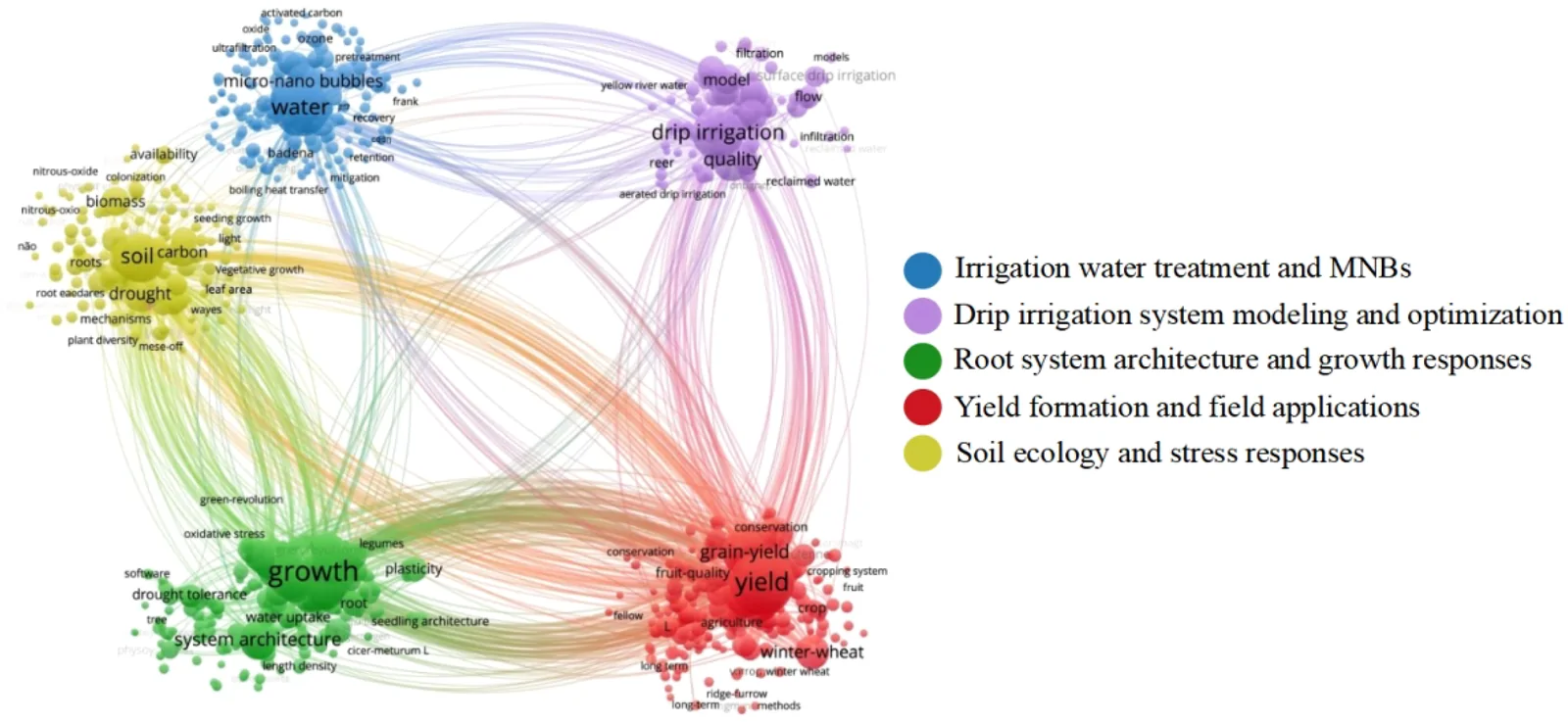
Keyword co-occurrence network of interdisciplinary research topics.

## 2 Root-zone water–air competition and oxygen transport regulation under SDI

### 2.1 Three-stage evolution of SDI technological paradigms

The trajectory of SDI engineering traces a structural paradigm shift (**Figure 3**) from single-liquid-phase delivery to coordinated multiphase regulation, and from passively accommodating environmental constraints toward actively directing engineering variables.

**Figure 3:**
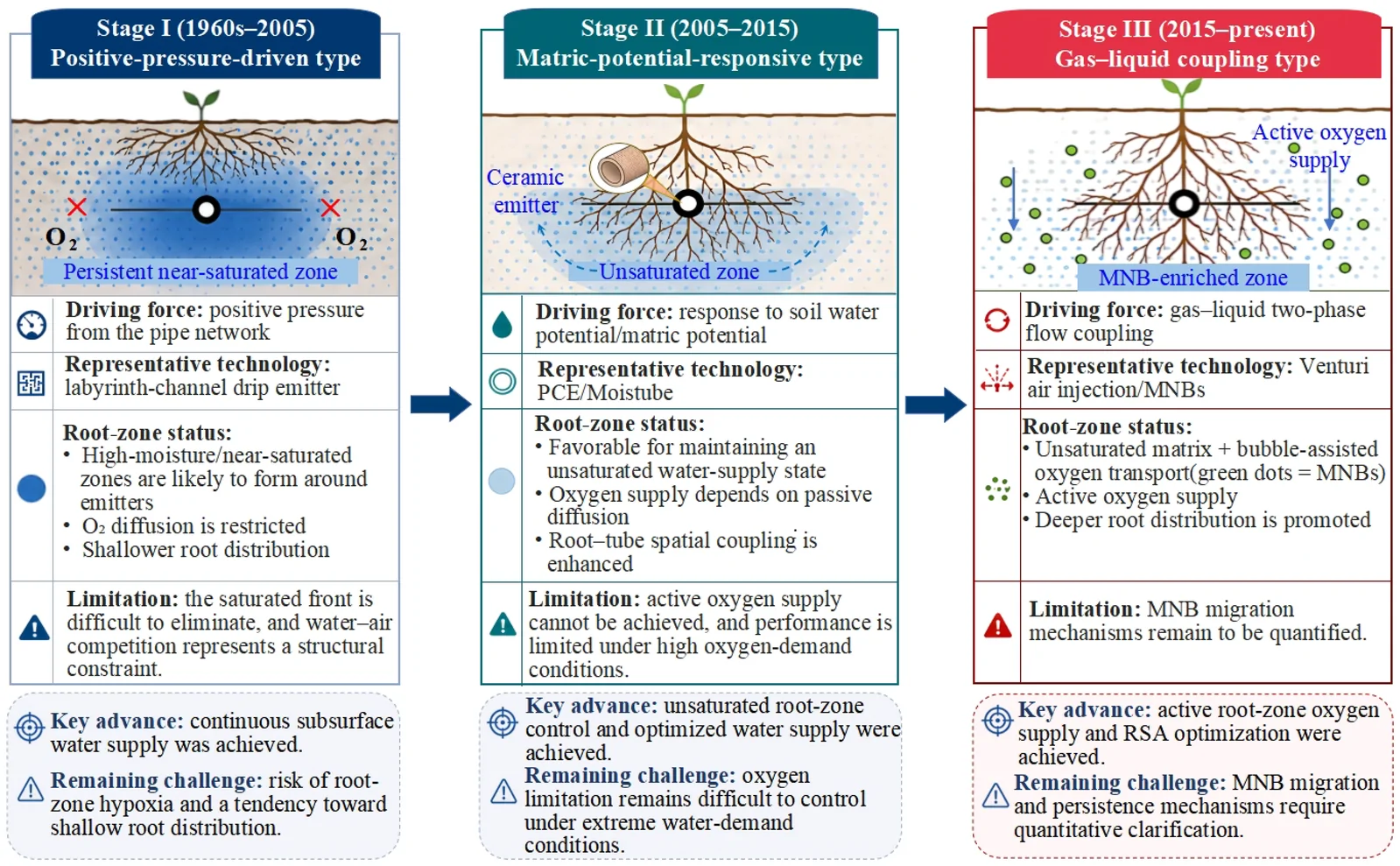
Engineering evolution of SDI technologies from the perspective of root-zone oxygen regulation. Main literature sources: Camp (1998); Lamm and Trooien (2003); Bhattarai et al. (2005, 2006); Ben-Noah and Friedman (2016, 2018); Cai et al. (2017, 2019, 2021); Kanda et al. (2018, 2020); Zhang et al. (2022); Zheng et al. (2025c); Li et al. (2025a).

In the first stage (**Figure 3**), the pressurized pipeline network achieved continuous liquid-phase transportation. The cost lies at the structural level: the moist front expands around the dripper, forming a locally saturated or high water content area, and the occupation of water gradually reduces the gas connectivity (Bajpai and Kaushal, 2020). At this point, the root system area extends beyond the end point of liquid-phase transport. The resulting hypoxic stress is not directly regulated by oxygen supply, but is indirectly managed by adjusting the drip depth and irrigation intermittency (Bhattarai et al., 2005; Lamm et al., 2021). In the second stage, water release is mainly driven by the applied positive pressure and gradually shifts to be regulated by the suction of the soil matrix, which helps to limit the range of the saturated area around the dripper (Cai et al., 2019; Kanda et al., 2018), while clay-based systems may promote local hyperplasia of roots along porous surfaces (Mahler, 2024). However, oxygen is still transported through passive diffusion at the atmospheric - soil interface. Under high transpiration demands, hypoxic stress is mainly alleviated through temporal supply redistribution rather than direct oxygen supply at spatial demand sites. The third stage redefines oxygen from a background limiting factor as a component that can be introduced and regulated in principle through irrigation water flow. This regulatory objective expands the scope of the coordinated management of water, oxygen and nutrients (Bhattarai et al., 2006; Du et al., 2018).

Field data from sandy clay loams are consistent with this interpretation. Compared to surface drip irrigation, air-injected SDI increased maize leaf area by 47.7% and yield by 37.78% and 38.46% over two consecutive seasons; relative to conventional SDI, the same treatment added approximately 12% (Abuarab et al., 2013). Long-term field evidence is also available for oxygated cotton in a heavy-clay Vertosol. Over seven seasons, oxygation increased lint yield from 182.7 to 200.3 gm^−2^, corresponding to a 10% average increase, while the gross production water-use index increased by 7% (Pendergast et al., 2013). These results indicate that subsurface air supply can alleviate root-zone oxygen limitation in the field, although the yield response varied with soil texture, crop species, and irrigation management. However, the outcome here predominantly reflects the agronomic crop-level responses to air-injected SDI, whereas the migration, retention, oxygen-release behavior, field-scale transfer, and scaling-up mechanisms of MNBs in heterogeneous soils remain poorly quantified. Therefore, crop-level agronomic responses cannot yet be regarded as direct evidence of MNB transport or root-surface oxygen delivery, and further validation studies are warranted. Despite two decades of research, aerated SDI has not yet achieved widespread commercial adoption. Several barriers account for this limited adoption: (i) the capital and energy costs of MNB generators and Venturi injectors are not offset by yield gains in low-value staple crops under current commodity prices; (ii) the long-term effects of aeration on emitter clogging remain uncertain and may vary with water quality and operating conditions; (iii) field-scale uniformity of bubble distribution along drip laterals has not been reliably demonstrated; and (iv) standardized design criteria linking aeration rate, soil type, and crop oxygen demand are still lacking (Balea et al., 2026; Ben-Noah et al., 2021; Li et al., 2025a). These constraints explain why the technology has not yet achieved widespread commercial adoption despite consistent positive results under controlled conditions. **Table 1** presents the three developmental stages across several dimensions.

**Table 1:** Multidimensional comparison of three types of SDI technology.

Comparison aspect	Positive pressure driven type (SDI)	Matric potential responsive type (PCE/Moistube)	Gas and liquid coupled type (aerated SDI/MNBW)
Engineering positioning	Centered on pipe network pressure and emitter energy dissipation to achieve a stable subsurface water supply.	Centered on low-pressure head, material permeability, and feedback from soil water potential to achieve a slow release water supply.	Centered on the coordination between SDI and active oxygen supply to achieve coupled regulation of water and air in the root zone.
Hydraulic characteristics	For emitters without pressure compensation, the flow regime exponent x can increase from 0.47 to 0.53 with operating years, thus inducing degradation of the turbulent flow.	The pressure and discharge relationship of PCE is approximately linear, with x ≈ 1.0. The discharge is sensitive to pipe network pressure fluctuations, making field-scale uniformity difficult to ensure.	Aeration modifies the flow field structure inside the flow path, expands the area of the high-velocity mainstream region, and affects hydraulic energy dissipation characteristics.
Water and air status in the root zone	Zones with high water content or near saturation are likely to form around emitters, restricting O_2_ diffusion.	An unsaturated slow-release water supply state is maintained, and oxygen supply mainly depends on the passive diffusion of pore gas.	Aerated water or MNBW increases DO in irrigation water and enhances the potential for oxygen supply in the root zone.
Quantitative performance reference points and evidence basis	A global meta-analysis (109 publications, 984 paired comparisons) reported that, relative to surface drip irrigation, SDI increased crop yield by an average of 5.39% and irrigation water productivity (IWP) by 6.75% (Wang et al., 2022). For emitters without pressure compensation, the flow regime exponent x increased from 0.47 to 0.53 over operating years, indicating progressive hydraulic degradation (Li et al., 2025b).	The pressure–flow relationship of PCE is approximately linear (x ≈ 1.0); no large-scale pooled estimate of yield or water productivity specific to this stage is currently available in the evidence base, which constitutes a recognized gap rather than an absence of effect.	Average increases of 19.3% in crop yield and 17.9% in WUE under aerated irrigation relative to controls (Du et al., 2018). In a separate short-term emitter study, aeration increased the average particle passage rate by 4–10 percentage points across three emitter types (Wang et al., 2025).
Operational risks	Clogging mainly occurs in emitter flow paths and outlets.	Low flow velocity and membrane pore structures are easily affected by suspended particles, dissolved solids, and biofilms.	A long-term oxygen-enriched environment may induce biofilm proliferation and chemical precipitation inside flow paths.
Application boundaries	Zones with high water content near emitters are difficult to avoid, and competition between water and air is a structural contradiction.	Active oxygen supplementation cannot be achieved, and the regulation capacity is limited under high transpiration conditions or in soils prone to hypoxia.	The migration, retention, dissolution, and root response chain of MNBW in unsaturated pores still requires quantitative analysis.

(1) The quantitative values listed under each stage are drawn from studies with different comparators and experimental conditions; they serve as reference points rather than directly comparable constants across stages. (2) x represents the flow regime index, namely the exponent in the power function relationship between emitter discharge Q and operating pressure head H, expressed as Q = kHˣ. Quantitative values for each stage derive from studies differing in comparators, response variables, and duration, and serve as performance reference points rather than directly comparable constants.

Main data sources: Lamm and Trooien (2003); Kanda et al. (2018); Du et al. (2018); Cai et al. (2019); Wang et al. (2022); Wang et al. (2025); Li et al. (2025b); Wang et al. (2026b).

### 2.2 Water–air phase competition and constraints on oxygen diffusion under point-source infiltration

By installing point source drippers for water supply, positive pressure subsurface drip irrigation (SDI) can reduce surface evaporation (Camp, 1998; Lamm et al., 2021); However, the soil around these drippers is highly prone to having a high water content or approaching saturation. The main influence is reflected in the aspect of oxygen. The diffusion coefficient of oxygen in the liquid phase is only about 10^-4^ of that in the gaseous phase. Therefore, the effective oxygen diffusion rate in the root zone will be significantly reduced (Ben-Noah and Friedman, 2018; Bhattarai et al., 2005), thereby altering root respiration and energy metabolism (Drew, 1997). This relationship is based on an established principle of soil physics: effective soil gas diffusivity increases with air-filled porosity, while pore structure and connectivity further modify this relationship (Penman, 1940; Millington and Quirk, 1961; Moldrup et al., 2000a, 2000b). Meanwhile, bulk density, aggregate size and substrate suction jointly regulate oxygen diffusion rate, REDOX potential and root elongation (Grable and Siemer, 1968). Numerical simulations have shown that soil water content around buried emitters is spatially heterogeneous under SDI, resulting in spatially uneven water–air constraints (Wang et al., 2013).Moreover, the wetted soil volume around a point-source emitter does not constitute a spatially uniform root-accessible domain; roots were concentrated near the periphery of the wetted volume and were sparse in its center, in association with spatial variation in oxygen diffusion rate (Silberbush et al., 1979). Field studies in Australian heavy clay Vertisols show that SDI can induce temporal and spatial root zone hypoxia after irrigation. In cotton, oxygation improved root development, yield, and water use efficiency under these conditions (Pendergast et al., 2013). In soybean, chickpea, and pumpkin, hypoxic limitations became more pronounced as emitter depth increased from 5 to 35 cm, while oxygation improved root activity and stomatal conductance (Bhattarai et al., 2008). These results indicate that the root zone oxygen environment is a long-underestimated constraint on WUE (Nair et al., 2013). However, improved soil oxygen concentration does not necessarily translate into significant growth, yield, or quality benefits. In a citrus orchard study, air injection through SDI raised soil O_2_ concentration but did not produce statistically significant improvements in tree growth, fruit yield, or fruit quality (Ben-Noah et al., 2021), indicating that the agronomic response to enhanced aeration is context-dependent and cannot be generalized from a single crop or soil type.

Matric-potential-responsive systems help moderate near-saturation fronts and promote root proliferation near the root–tube interface (Cai et al., 2019; Kanda et al., 2018; Mahler, 2024). However, these benefits remain limited. According to a recent study by Namdarian et al. (2024), the burial depth of an emitter can impact soil moisture and salinity distribution without a significant effect on yield. A global meta-analysis further showed that suitable burial depth varied among crop groups, with 30 to 45 cm being more suitable for solanaceous vegetables and 15 to 30 cm for legume crops (Zhang et al., 2025b). These studies did not directly assess the oxygen status in the root zone. The risk of hypoxia depends on air-filled porosity and the connectivity of oxygen diffusion paths, both of which fluctuate with the irrigation cycle. When the transpiration demand is high, the oxygen consumed by the root system must be replenished through passive diffusion by the remaining gas filling the pores, which makes the geometry of the pore network a potential limiting factor. Based on the production function relationship between irrigation volume and corn yield under SDI, when the irrigation volume approaches the level of sufficient irrigation water, the marginal yield gain tends to decline. This model indicates that marginal diminishing returns may occur solely through liquid-phase regulation (Irmak, 2024). In the first two generations of SDI development, the engineering goals focused on water transport, the formation of wetting patterns and the hydraulic characteristics of drippers. However, the decline in gas connectivity and oxygen diffusion in the moist area closest to the dripper has always been a secondary concern. Over time, operational risks exacerbated this situation. Flow channel blockage, particle deposition under negative pressure conditions, and the difficulty in reaching the buried drip irrigation pipelines complicate inspection and maintenance, gradually reducing system reliability (Li et al., 2025b). These limitations have prompted the engineering objective to shift from liquid-phase water transport to pore-scale multiphase flow management, providing a theoretical basis for gas-liquid coupling regulation.

### 2.3 MNB-mediated gas–liquid coupled mass transfer and multiphase flow coordination

According to ISO 20480-1:2017, MNBs include microbubbles (1–100 μm in diameter) and ultrafine bubbles (<1 μm). Owing to their large specific surface area, high internal pressure, and negative zeta potential (generally<−30 mV), MNBs facilitate gas-phase dispersion through soil pore networks and serve as microscale oxygen reservoirs, thereby reducing the spatial competition between liquid-phase water delivery and gas-phase oxygen transport (Chen et al., 2025a; Zheng et al., 2025a). However, these advantages have been demonstrated primarily in laboratory experiments and under controlled conditions and should not be extrapolated directly to heterogeneous field soils (Chen et al., 2025a; Borsky et al., 2024). In saturated model columns, nanobubble mobility and retention varied with medium particle size, pH, ionic strength, and cation valence, whereas wettability had little effect; validation in field soils remains necessary (Borsky et al., 2024).Field studies have shown that drip irrigation with nanobubble oxygenated treated wastewater can improve soil aeration (Baram et al., 2022).In numerical simulations of three emitter designs, aeration increased particle passing rates by 10, 9, and 4 percentage points, respectively, with a mean increase of approximately 7.7 percentage points (Wang et al., 2025).However, nonuniform air delivery among emitters along drip laterals remains an operational limitation (Torabi et al., 2014).

Experimental evidence indicates that MNBW irrigation can provide greater spatiotemporal optimization of crop growth than conventional microbubble water irrigation under controlled conditions. A meta-analysis reported that MNBW irrigation increased crop yield and root dry weight by an average of 13.55% and 27.21%, respectively, compared with controls without MNBW irrigation (Jiao et al., 2026a).Similarly, studies by Ouyang et al. (2023) and Ouyang et al. (2025) on cucumber demonstrated that MNBW irrigation improved yield. In the DO × biochar experiment, yields were 1.72%–44.23% higher than CK; therefore, the maximum response cannot be attributed to oxygenation alone (Ouyang et al., 2025).Nevertheless, crop responses vary among species. Changes in the strawberry rhizosphere bacterial community were associated with improved yield and fruit quality under SDI supplied with MNBW (Zheng et al., 2025c). Similarly, Robles et al. (2026) reported that oxyfertigation with H_2_O_2_ increased irrigation water DO and soil oxygen diffusion rate in a mature citrus orchard. The use of aerated SDI altered soil water and salt distribution; under aerated conditions, reducing irrigation by 20% maintained grain yield and increased harvest index and irrigation water productivity relative to full irrigation (Li et al., 2026). Field studies have also reported that MNB irrigation can alter soil microbial communities and improve soil fertility (Zhou et al., 2020; Wang et al., 2024). Zhu et al. (2019a) reported that aerated irrigation increased soil respiration and microbial abundance. In addition, Zhang et al. (2022) showed that aerated irrigation activated the expression of indole-3-acetic acid (IAA) signaling-related and YUCCA auxin biosynthesis genes during tomato cultivation, thereby promoting root growth. Wang et al. (2026a) further reported that MNB oxygenation increased plant phosphorus content, soil enzyme activities, and microbial richness, whereas oxygenation × phosphorus interactions were significant for plant phosphorus content and soil phosphorus in the upper 0–50 cm, but not for enzyme activities or microbial richness. However, across these studies, the specific contribution of dissolved oxygen relative to other processes associated with MNBs was not consistently isolated.

Overall, aerated irrigation is associated with an increase in soil fertility indicators and crop yields, although the causal path from dissolved oxygen to yield has not been fully elucidated. Therefore, the root zone becomes a controllable multiphase reaction domain rather than merely a passive endpoint for liquid-phase water transport. A proposed regulatory principle for gas-liquid coupled subsurface drip irrigation (SDI) is to coordinate the supply of oxygen, water and nutrients. However, the optimal sequence and relative time still vary by crop and soil and have not yet been established as a universal procedure. This cross-dimensional research framework is shown in **Figure 4**.

**Figure 4:**
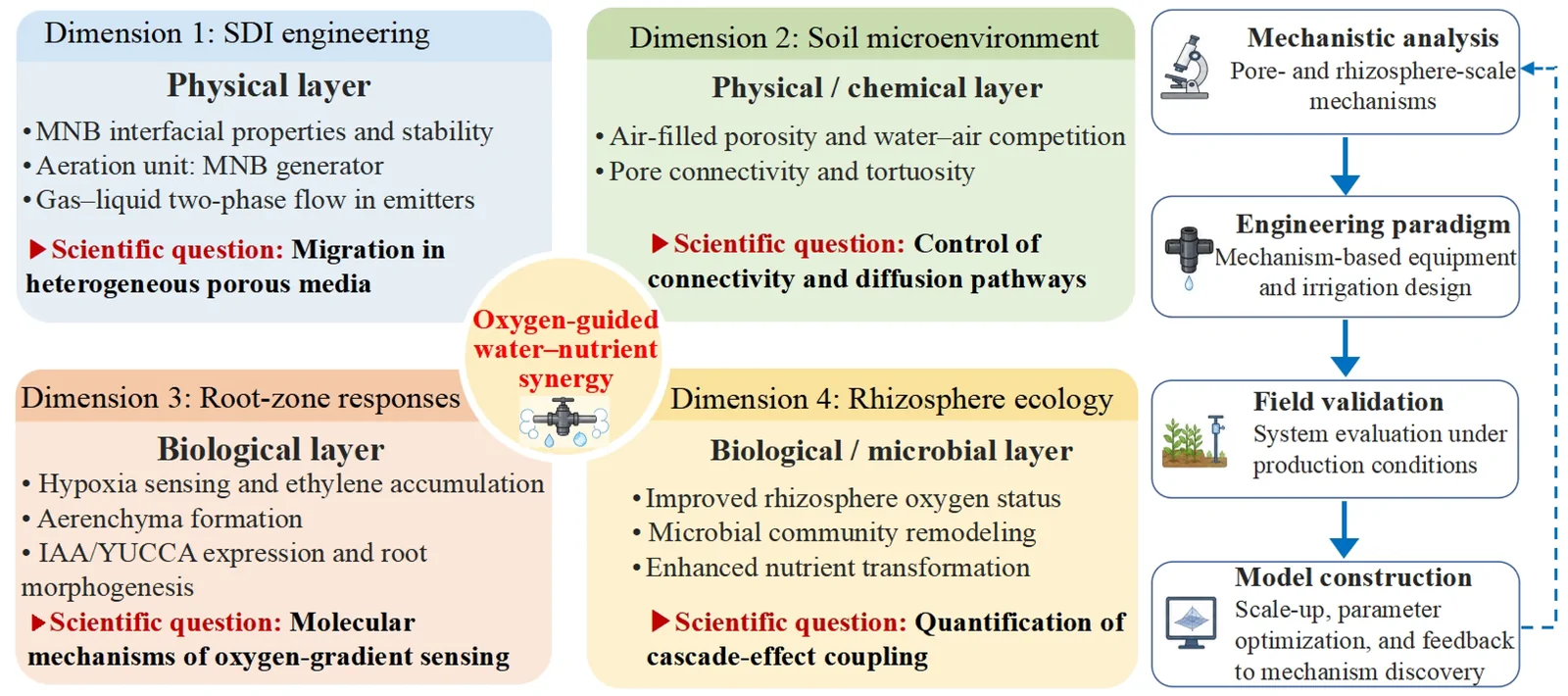
Multidimensional framework for gas–liquid coupled SDI.

The framework shown in **Figure 4** integrates multiple levels - liquid-gas phase competition and oxygen diffusion constraints, MnB-mediated mass transfer enhancement, and rhizosphere microbial community remodeling - into a coupled system, covering four dimensions: engineering technology, soil physics, root biology, and rhizosphere ecology. There are still several research gaps: The migration and oxygen release behavior of MNB in porous media and its effective mass transfer distance remain unclear; The regulatory mechanism of oxygen diffusion path connectivity in unsaturated soil remains to be clarified. The hormone signal transduction network on which the root system senses the oxygen gradient remains unknown. The microbial and nutrient stratification linkage effects triggered by the oxygen environment in the root zone also remain to be revealed. From an economic perspective, the capital investment, operational energy consumption, maintenance requirements of MNB generation equipment, as well as the risk of accelerated clogging of irrigation devices under oxygen-rich conditions, all need to be weighed against agronomic benefits. At present, the full life cycle cost analysis of aerated drip irrigation (SDI) and traditional drip irrigation (SDI) under different planting systems is still relatively scarce, and the conditions for the economic rationality of additional investment have not yet been systematically defined (Balea et al., 2026). Addressing these gaps is critical for advancing gas–liquid coupled SDI from empirical observation toward mechanistic understanding and economically viable field deployment.

## 3 Mechanisms of root system architecture remodeling driven by water–nutrient–oxygen coupling from the perspective of root foraging costs

### 3.1 Changes in root foraging costs

In most studies on subsurface drip irrigation (SDI), the root zone is mainly regarded as the site for water transport and redistribution. The research focuses on soil water movement, moist body development and hydrothermal transport processes in the emitters - soil system (Kandelous et al., 2011; Wang et al., 2013. In contrast, the root system, as an organ that actively regulates its own resource acquisition, is rarely involved in this feedback mechanism. However, under SDI conditions, the micro-domain with water enrichment but oxygen limitation may become a key area where root metabolic requirements, local resource availability and biomass allocation are simultaneously restricted. Under the cost-benefit analysis framework of the optimal foraging theory, continuous root proliferation is expected to occur only when the marginal benefit of resource acquisition exceeds the marginal cost of constructing and maintaining the root system (McNickle et al., 2009; McNickle and Cahill, 2009). This logic stems from the marginal value theorem: When the local resource acquisition rate is lower than the average level of the habitat, foragers should leave the depleting resource patches (MacArthur and Pianka, 1966; Charnov, 1976). After the tissue is formed, the growth of the root system is mainly irreversible. The established functional root segments are spatially fixed and cannot migrate from the diminishing patches (Renton et al., 2025). Local nutrient enrichment can trigger directed proliferation of the root system (Drew and Saker, 1975), but the same cost-benefit logic also applies to oxygen: if the metabolic cost of maintaining the root system in hypoxic plaques exceeds the benefits obtained from water and nutrient absorption, subsequent growth investment should be transferred to other areas.

Hypoxia directly increases these costs. When irrigation water fills soil pores and reduces gas-phase connectivity, oxygen diffusion is constrained, promoting increased root dependence on glycolysis and fermentation rather than on aerobic respiration, thereby notably lowering the ATP production efficiency (António et al., 2016; van Veen et al., 2025). The metabolic penalty is substantial: under anoxia, glycolysis yields a net 2 mol ATP per mol glucose (Drew, 1997), and this reduced energy supply constrains both ion uptake and cell expansion. The consequences are twofold: root nutrient and water uptake and elongation weaken (Drew, 1992; Drew, 1997), and water- and nutrient-enriched zones become “high-cost traps.” Even where water and nutrients are relatively abundant, the physiological costs associated with oxygen limitation may constrain sustained root growth (Bhattarai et al., 2004), while new growth may be redirected laterally toward better aerated regions (Eysholdt-Derzsó and Sauter, 2017). In heavy clay soils, spatiotemporally heterogeneous hypoxia produced by persistent wetting fronts under SDI limits root development and ultimately yield potential (Pendergast et al., 2013). However, mechanical impedance and soil respiration may affect root growth and soil oxygen status, respectively (Grable and Siemer, 1968); therefore, the observed architectural responses cannot be attributed to oxygen limitation alone without factorial separation of these factors.

Thus, aerated irrigation involves delivering oxygen supplementation to the core of the wetted soil volume, which may partially restore aerobic respiration, reduces carbon costs, and reactivates the proliferative potential of the roots. Direct evidence comes from greenhouse tomato experiments, where cyclic aerated SDI increased root IAA content by approximately 2.3-fold and upregulated auxin biosynthesis genes, such as YUCCA (Zhang et al., 2022). This treatment also promoted deeper fine root proliferation, shifting them from a shallow concentration to a high-density distribution in the mid-to-deep layers (Zhang et al., 2023b). These architectural changes are consistent with the hypothesis that aeration reopens an RSA plasticity window previously restricted by hypoxia, with the proposed trigger being a reduction in root-foraging metabolic costs. However, this interpretation remains correlative; the tomato experiments did not isolate oxygen from concurrent changes in redox status, nutrient availability, and microbial activity, and the results have not been replicated under field conditions with heterogeneous soils.

### 3.2 Oxygen sensing, signal integration, and root foraging cost signals

How does the root system convert local oxygen gradients into structural regulation instructions? Its molecular interface involves multiple signaling pathways - upstream oxygen perception, reactive oxygen species (ROS), nitric oxide (NO), and downstream ethylene-auxin crosstalk - these pathways collectively link metabolic costs to spatial investment decisions. Under SDI conditions, the gradients of water, nutrients and oxygen overlap in the root zone. The root system perceives these gradients through three different pathways: water orientation, nutrient foraging response and oxygen orientation. Occasionally, these three exhibit inconsistencies (Karlova et al., 2021; Morris et al., 2017). Giehl and von Wiren (2014) characterized the plasticity of root foraging in heterogeneous nutrient plaques, while Bao et al. (2014) clarified the molecular basis for the formation of lateral root water patterns. Under normoxic conditions, the N-terminal rule pathway of the ubiquitin-proteasome system targets the seventh group of ethylene response factors (ERF-VIIs) and promotes their degradation. When the oxygen level decreases, ERF-VIIs accumulate and activate hypoxia response genes (Licausi et al., 2011; Gibbs et al., 2011). This oxygen-sensing mechanism provides a direct molecular link between local oxygen partial pressure and transcriptional reprogramming. Meanwhile, hypoxia induces the expression of RBOHH and the production of ROS in the root cortex cells of rice, and the resulting ROS participate in programmed cell death during the formation of lysigenous aerenchyma (Yamauchi et al., 2017); During adventitious root development, NO is involved in root primordia initiation, while ethylene promotes root protrusion from the epidermis (Steffens and Rasmussen, 2016). These channels do not operate in isolation; Its relative contribution may depend on the severity and duration of hypoxia, the stage of root development, and species-specific anatomical structure.

Under hypoxia, primary root growth is inhibited (Eysholdt-Derzsó et al., 2024), while altered auxin transport and signaling redirect its growth toward more horizontal trajectories (Eysholdt-Derzsó and Sauter, 2017). Under aerated SDI, the same crosstalk operates as a two-way translation of cost signals. When oxygen is sufficient, ethylene signaling is suppressed; auxin-dominated hydrotropic and nutrient-foraging signals proceed normally and facilitate root proliferation toward the core of the wetted soil volume. When oxygen is deficient, accumulated ethylene disrupts auxin-mediated growth regulation and directs the roots into an avoidance architecture. This explains why, under SDI, roots may remain shallow or shift spatially, even where water and nutrients are abundant near the emitters (Bhattarai et al., 2008; Zang et al., 2018; Zhu et al., 2019b). A similar pattern has been observed under salt stress, where roots avoid high-salinity patches through architectural plasticity (Shelden and Munns, 2023); thus, spatial avoidance appears to be a shared response across stress contexts, with oxygen-mediated cost regulation being one of its candidate mechanisms. It must be emphasized that the evidence for these signaling pathways derives predominantly from waterlogging, submergence, and controlled-atmosphere studies in model species (Arabidopsis, rice) or crop seedlings under laboratory conditions (Licausi et al., 2011; Eysholdt-Derzsó and Sauter, 2017; Yamauchi et al., 2017). Whether the localized, transient hypoxic microdomains generated around SDI emitters—which differ in spatial extent, duration, and severity from whole-root-zone flooding—can activate the same signaling cascades remains an untested extrapolation. Validation requires *in situ* measurements of ERF-VII accumulation, ROS gradients, and hormone concentrations in roots growing within the oxygen-gradient field of operating SDI emitters.

### 3.3 Three root architectural response modes driven by cost–benefit trade-offs

The balance between root foraging costs and benefits categorizes architectural responses under different water–nutrient–oxygen coupling conditions into three conceptual modes (**Table 2**). These modes represent idealized endpoints of a continuous spectrum; field conditions typically produce intermediate or mixed responses, and the boundaries between modes depend on the relative magnitudes of oxygen limitation, nutrient availability, and mechanical impedance at a given site.

**Table 2:** Three RSA response modes under coupled water, nutrient, and oxygen conditions.

Comparison aspect	I. Spatial avoidance	II. Local proliferation dilemma	III. Whole domain exploration
Resource configuration	High water and low oxygen	High water, high nutrient, and low oxygen	High water, high nutrient, and high oxygen
Cost and benefit condition	Costs are too high to be compensated by returns	Costs and benefits remain in a stalemate	Costs decline and returns are maximized
Architectural response	Shallow distribution and lateral displacement	Local high-density proliferation with limited depth	Expansion toward the core zone of the wetted soil volume and middle to deep soil layers
Representative evidence	Bhattarai et al. (2008); Pendergast et al. (2013); Zang et al. (2018)	Ouyang et al. (2021); Ouyang et al. (2023); Qian et al. (2025)	Zhang et al. (2022); Li et al. (2024)
Evidence type and limitations	Field and pot studies under SDI with and without oxygation; oxygen effects were assessed; limitations remain associated with emitter depth and soil texture	Greenhouse studies with controlled DO levels and uniform nutrient supply, supplemented by review evidence; the oxygen-nutrient interaction in spatial RSA has not been factorially isolated	Greenhouse and field experiments with controlled aeration; field-scale replication of this spatial RSA pattern and its long-term stability have not yet been demonstrated

Mode I is referred to as spatial avoidance. This can mitigate local hypoxic stress but at the cost of sustained exploitation of water-enriched zones. Under conventional SDI, “water-rich but oxygen-limited” traps tend to form near emitters. Water accumulation reduces gas-phase porosity and restricts oxygen diffusion, making zones favorable for water acquisition oxygen-limited (Bhattarai et al., 2008; Bhattarai et al., 2005). Hypoxic stress suppresses root aerobic metabolism and uptake and may reduce or even override primary root gravitropic growth, inducing lateral or shallower development. At this point, aerotropic demand can outweigh hydrotropic and nutrient-foraging signals, and RSA remodeling is driven toward avoidance (Drew, 1997; Karlova et al., 2021).

Mode II is the local proliferation dilemma. Tropism toward water- and nutrient-enriched zones drives proliferation; however, under oxygen limitation, proliferation becomes constrained by a cost–benefit trade-off. When nutrient gradients overlap with the soil water field, nutrient foraging responses drive local proliferation in nutrient-rich patches (Cheng et al., 2016; Hodge, 2004; Qian et al., 2025). Dense root clusters lead to intense respiration, which in turn exacerbates hypoxia in the root zone and forms a self-reinforcing negative feedback loop characterized by high water and nutrient availability as well as oxygen limitation. Under oxygenated irrigation conditions, soil microbial biomass carbon and nitrogen, soil respiration and other related indicators increased significantly (Lei et al., 2022; Ouyang et al., 2021; Ouyang et al., 2023, indicated that simple water-nutrient coupling might not be sufficient to completely alleviate oxygen limitation in the root zone. Under the coupling condition of water, nutrients and oxygen, the synergistic improvement of soil aeration and water and fertilizer utilization efficiency depends on the reasonable regulation of oxygen supply. Insufficient oxygen supply or poor operating conditions may weaken these benefits (Junejo et al., 2025; Liu et al., 2023).

Mode III represents the exploration of the entire root system domain. Oxygen, as a potential third regulatory variable, can alleviate hypoxia stress and promote more extensive root expansion at an appropriate supply level. Meanwhile, it coordinates the spatial distribution, physiological functions and rhizosphere microecology of the root system (Li et al., 2024; Yu et al., 2024; Zhang et al., 2022). The reported benefits cover a variety of crops, including improved cotton growth, yield and water use efficiency (Bian et al., 2025; Zhang et al., 2025c); Enhance root vitality, rhizosphere microbial activation and yield formation of perennial forage (Cao et al., 2025); And it promotes a denser distribution of the root system of shallow-rooted fruit trees and extends to the middle and deep soil (Zhang et al., 2026b; Zheng et al., 2025b).In tomatoes, an improved oxygen supply also notably increases root activity during flowering and fruit expansion (Li et al., 2024). When dissolved oxygen remains within an appropriate threshold (Ouyang et al., 2021), water–oxygen coupling can sustain gains in water and fertilizer-use efficiency, fruit yield, and fruit quality (Liu et al., 2019; Liu et al., 2024), and continuously cropped dryland maize has also demonstrated long-term yield improvement (Yu et al., 2024). However, these results should be interpreted with caution: most Mode III evidence comes from greenhouse or pot experiments with controlled aeration rates; whether the same architectural expansion occurs under field-scale heterogeneity, where MNB distribution along laterals is uneven and soil properties vary spatially, has not been confirmed.

Considering the three modes, progression is not a passive root adaptation to oxygen concentration but a proposed active reconfiguration of spatial investment under metabolic-cost constraints, from avoidance through constrained proliferation to whole-domain exploration. Reframing the problem from “oxygen concentration” to “foraging cost” similarly repositions the functional mechanism of aerated SDI: the proposed key mechanism is not oxygen supply per se but the regulation of root foraging costs; this interpretation remains a working hypothesis that requires experimental separation of oxygen effects from nutrient, mechanical, and microbial confounders. Based on this (**Figure 5**), we propose a cross-scale oxygen-mediated mechanistic framework for foraging cost-driven RSA remodeling.

**Figure 5:**
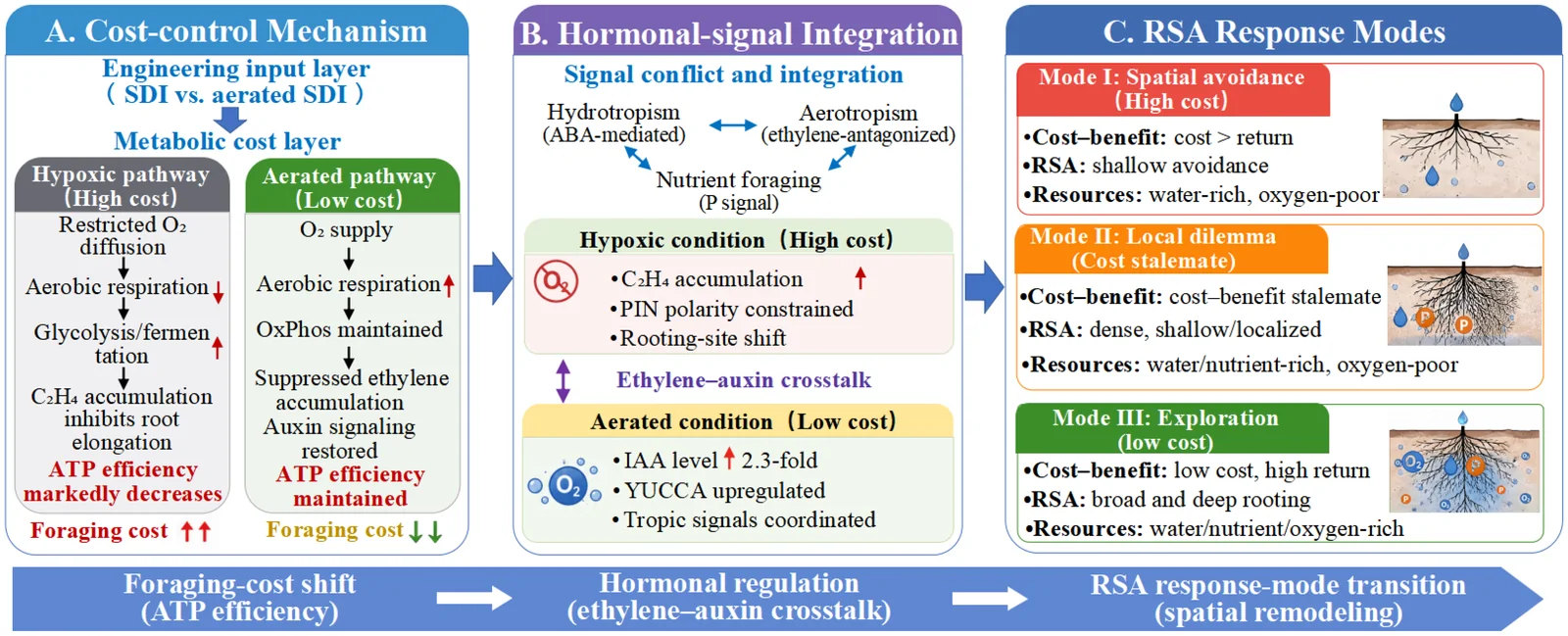
Mechanistic framework for oxygen-mediated root system architecture remodeling. Main literature sources: Bhattarai et al. (2008); Pendergast et al. (2013); António et al. (2016); Eysholdt-Derzsó and Sauter (2017); Morris et al. (2017); Ouyang et al. (2021); Karlova et al. (2021); Lei et al. (2022); Zhang et al. (2022); Ouyang et al. (2023); Eysholdt-Derzsó et al. (2024); Li et al. (2024); Yu et al. (2024); van Veen et al. (2025).

**Figure 5** presents the framework organized into three modules. Module A traces how hypoxia reduces ATP production efficiency and transforms water-enriched zones into “high-cost traps.” Module B shows how oxygen sensing, ROS/NO signaling, and ethylene–auxin crosstalk translates metabolic cost signals into architectural cues. Module C maps these mechanisms onto three spatial modes of the root architectural response. Together, they indicate a practical shift: the SDI emitter placement criterion can move from conventional water coverage toward the root-foraging-accessible domain.

### 3.4 Conceptualization of the root foraging-accessible domain

The wetted soil volume around the emitter should not be equal to the root-foraging-accessible space. While irrigation increases the local soil water content, it can simultaneously reduce gas-phase connectivity and oxygen diffusion. Therefore, a zone may exceed the minimum water threshold for effective uptake; however, after its local oxygen partial pressure decreases below the aerobic-metabolism threshold, it transitions into a “high-cost microdomain” for root growth, in which root respiration, water absorption, and nutrient acquisition are all constrained by oxygen limitation (Beegum et al., 2023; Pragg et al., 2024; Chen et al., 2025b).The concept of jointly constrained water and air availability in the root zone has precedent: Leão et al. (2005) described the least limiting water range (LLWR) as the range of soil water content within which limitations to plant growth associated with water potential, aeration, and mechanical resistance are minimized. FAD extends this logic by treating oxygen availability as a spatially explicit, time-varying constraint specific to the emitter-influenced domain, rather than as a single bulk-soil threshold. The critical partial pressure of oxygen (pcrit) required to maintain aerobic respiration in root systems is not a universal constant. Rather, it depends on parameters such as species, root age, tissue anatomical structure (cortical thickness, degree of ventilation tissue development), temperature, respiratory requirements and measurement methods (comparison between intact root system and isolated tissue, comparison between steady-state and transient exposure) (Armstrong and Gaynard, 1976; Saglio et al., 1984; Armstrong et al., 2009. In the measurements of intact plants and intact root systems, the reported pcrit values were approximately 2–4 kPa (Armstrong and Gaynard, 1976; Armstrong et al., 2009); However, Saglio et al. (1988) used this range as the pretreatment condition for hypoxia rather than the measured pcrit value; Such values should be regarded as the reference magnitude under a specific experimental system, rather than the threshold for direct extrapolation across species, soil or field conditions (Bhattarai et al., 2005; Armstrong et al., 2009). Before applying this parameter to a specific system, local calibration is required - that is, matching the pcrit with the target crop, root development stage, soil temperature and measurement scheme. Therefore, we no longer equate the volume of the moist area with the available space of the Root system. Instead, we regard water accessibility and oxygen accessibility as parallel constraints and propose a conceptual framework - Root Foraging-Accessible Domain (FAD). FAD is defined in two forms: the general continuous suitability expression and the simplified binary threshold form that appears as a limit case. The general definition adopts continuous membership functions" to cite Equation 1:

(1) $$\Omega_{\text{FAD}}^{*}\left(t\right)=\left\{r\in \Omega_{\text{soil}}|w\left(r,t\right)\geq w_{0}\right\}$$

where *w(r, t)* is the integrated foraging suitability jointly determined by water and oxygen suitability, and *w*_0_ is the threshold for classifying FAD. The function *w*(*r*, *t*) maps each location to a value between 0 (completely unsuitable) and 1 (fully suitable), reflecting the graded nature of both water and oxygen availability. Its specific functional form may be derived from measured soil water content, oxygen partial pressure, root physiological response curves, or coupled water–gas simulations; a fixed form is not prescribed here because the appropriate parameterization will depend on the crop, soil, and measurement resolution available. The binary-threshold form is a special case that emerges when the suitability function is replaced by step functions—equivalent to setting the Hill coefficient to infinity in a sigmoidal formulation as shown in Equation 2:

(2) $$\Omega_{\text{FAD}}(t)=\left\{r\in \Omega_{\text{soil}}|\theta (r,t)\geq \theta_{\min}(s,\sigma )\wedge p_{O_{2\;}}(r,t)\geq p_{\text{crit }}(s,a,T,\epsilon )\right\}$$

where Ω_FAD_(t) represents the root foraging-accessible domain at time *t*; Ω_soil_ is the predefined soil evaluation domain; *r* is the spatial position vector; *θ(r, t)* is the volumetric soil water content; *θ*_min_*(s,σ)* is the lower threshold of effective water availability, where σ represents soil hydraulic conditions $p_{O_{2}}\left(r,t\right)$ represents the soil oxygen partial pressure; and *p_crit_(s,a,T,ϵ)* is the critical oxygen partial pressure, parameterized by crop species *s*, root age/anatomy *a*, temperature *T*, and measurement conditions *ϵ*. This binary form is operationally simpler but sacrifices the graded information that the continuous form retains; it is appropriate only when the transition between adequate and inadequate oxygen is abrupt relative to the spatial resolution of interest.

(3) $$\eta_{\text{FAD }}(t)=\frac{V_{\text{FAD}}(t)}{V_{\text{wet}}(t)},\;V_{\text{wet}}(t)>0$$

where:

(4) $$V_{\text{FAD}}(t)={∭}_{\Omega_{\text{FAD}}(t)}\text{dV}$$

(5) $$V_{\text{wet}}(t)={∭}_{\Omega_{\text{wet}}(t)}\text{dV},\;\Omega_{\text{wet}}(t)=\left\{r\in \Omega_{\text{soil}}|\theta (r,t)\geq \theta_{\min}(s,\sigma )\right\}$$

The volume integrals defining VFAD(t) and Vwet(t) are given in Equations 4, 5, respectively where V_FAD_(*t*) is the volume of the root foraging-accessible domain $\Omega_{\text{FAD}}(t)$ and V_wet_(*t*) is the volume of the wetted soil domain $\Omega_{\text{wet}}(t)$ satisfying the water-availability condition; both volume integrals are applied over the three-dimensional domain and expressed in m^3^, and Equation 3 is defined only when V_wet_ > 0. Thus, η_FAD_ measures the fraction of the wetted domain that simultaneously meets the water and oxygen availability constraints, distinguishing zones merely wetted by irrigation from those potentially available for effective root use. At this stage the FAD is understood as a working conceptual framework, a distinction that defines what remains to be measured. Its operationalization follows the sequence determined by its own definition. Firstly, it is necessary to conduct spatially resolved synchronous measurements of soil moisture content and oxygen partial pressure within the area affected by the dripper to ensure that the two constraints entering the FAD criterion are based on actual measurement rather than inference. Secondly, it is necessary to conduct specific calibration of *p*_crit_ for crops under representative field conditions - including soil type, temperature conditions and root development stage - and independently confirm that the calculated FAD boundary is consistent with the observed root distribution. Finally, sensitivity analysis should be conducted to determine which type of expression, continuous or binary, can better reflect the root response at the actual achievable measurement resolution. Before these steps are completed, *η*_FAD_ is most suitable as a diagnostic indicator for comparing irrigation scenarios rather than a normative design guideline.

## 4 Conclusions and future perspectives

### 4.1 Conclusions

SDI has inherent structural trade-offs in the soil pore space: it is quite challenging to simultaneously meet the supply of water and oxygen. This review holds that in the heterogeneous water-oxygen field generated by point source infiltration, the root response may not be fully explained merely by soil moisture distribution or root morphology. From the perspective of the optimal foraging theory, root plasticity under aerated irrigation is more appropriately understood as a spatial investment adjustment, which tracks the changes in foraging metabolic costs. The reconstruction framework proposed in this review lies precisely here: By integrating evidence from root ecology, plant oxygen signaling, and irrigation engineering, we reposition aerated SDI from a purely hydraulic intervention to a framework for managing the location and cost of root resource acquisition. The core proposition - that root zone oxygen availability not only serves as a growth promoter but also as a variable regulating the metabolic cost of resource acquisition - is proposed here as a testable mechanism hypothesis framework.

The existing evidence converges into a coherent mechanism along the path from oxygen perception and signal integration of the physical environment in the root zone to root configuration adjustment: Micro-nano bubbles (MNBs) can alleviate the limitations of water-rich and oxygen-poor conditions on root activity, reduce the physiological cost of foraging, and thereby reshape root configuration and rhizosphere microbial function by increasing the dissolved oxygen concentration around the root tip. We propose this as a mechanism-based explanation mainly based on correlation and dominated by controlled environmental evidence, rather than a closed causal chain. There are still two boundaries to be resolved: the spatiotemporal dynamics of irrigation-induced oxygen fluctuations and the field-scale behavior of MNBs in heterogeneous soil. By listing these as open-ended questions, this review transforms descriptive narratives into predictions that can be confirmed or refuted.

Oxygen mediation is not solely an environmental factor that promotes root growth; however, it is also a key variable regulating the cost and efficiency of root resource acquisition. Accordingly, the optimization target of SDI should transition from increasing soil water coverage alone to optimizing FAD. Additionally, aerated irrigation does not need to be defined solely by the synchronous supply of water, nutrients, and oxygen; however, it can be reframed around the interactions among roots, soil, water, nutrients, oxygen, and the microbiome, together with root behavioral regulation. This perspective may provide a more coherent theoretical basis for improving water and nutrient use efficiencies in irrigated agriculture.

### 4.2 Future research directions

Future studies should focus along three directions: microscopic mechanisms, cross-scale integration, and macroscale application, and, more specifically, across four levels: decision modeling of rhizosphere ecosystems, functional–structural plant models embedded with OFT, climate-adaptive resilience design of irrigation systems, and scale-up and translational pathways. The cost–benefit decision engine of the OFT serves as the central link, providing evidence from microscopic processes to macroscale regulation targets and supporting a smart root-zone regulation system based on a sensing–simulation–feedback loop (**Figure 6**).

**Figure 6:**
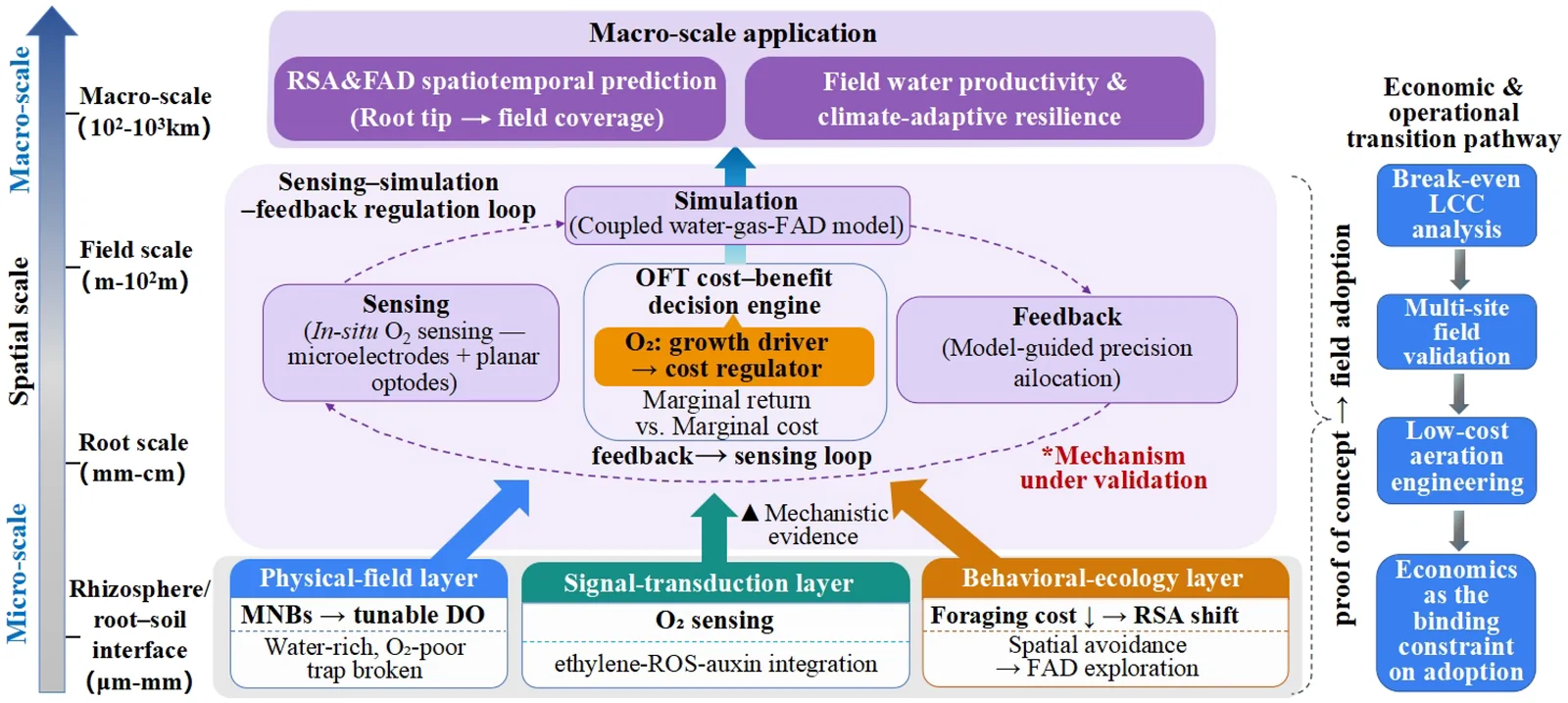
Cross-scale framework linking the oxygen-mediated foraging-cost hypothesis from the individual root tip to the field, and the sequence of tests and translational steps required to evaluate it.

#### 4.2.1 Establishing the causal role of oxygen

The most consequential open question is whether oxygen independently drives RSA remodeling or merely co-varies with the nutrient redistribution, redox shifts, microbial restructuring, and reduced mechanical impedance that accompany aeration. Establishing causality therefore requires experiments that manipulate oxygen availability while holding these factors constant—for example, split-root rhizotrons with independent gas-phase control in each compartment, or field plots in which inert-gas displacement generates matched-water, low-oxygen controls against MNB irrigation. Because irrigation-induced hypoxia fluctuates on diurnal, weekly, and seasonal timescales rather than remaining static, such designs must also resolve time: whether a brief hypoxic pulse of a few hours engages the same ERF-VII–ethylene–auxin cascade and architectural response as an oxygen deficit sustained over days to weeks is currently unknown, and can be addressed by pairing continuous root-tip oxygen microelectrode profiling with sequential harvests or non-destructive imaging. Within the same experiments, ^14^C pulse-labeling (Lucas et al., 2023) and three-dimensional root mesocosm phenotyping (Dowd et al., 2022) can test the metabolic prediction at the core of the hypothesis—that restored aeration lowers the carbon cost per unit root elongation—while rhizosphere metagenomic profiling can distinguish whether oxygen-driven shifts in exudate composition actively recruit microbial functional modules (Zhang et al., 2026a) or merely follow from altered root physiology.

#### 4.2.2 Making the FAD quantitative and spatially explicit

Turning the FAD from a diagnostic concept into a predictive tool depends on three linked advances, each within reach of existing methods. The critical oxygen pressure that bounds the domain must first be calibrated for intact roots under field-relevant temperatures, soil textures, and developmental stages, because the 2–4 kPa values reported in the literature were obtained under controlled conditions using different experimental approaches and cannot be assumed to hold *in situ*; oxygen microelectrodes and planar optodes deployed within the emitter-influenced zone (Ahkami et al., 2024) make this measurement feasible. These thresholds can then be embedded in coupled water–oxygen models: vadose-zone solvers such as HYDRUS-2D already resolve soil-water distribution around emitters, and linking them to gas-diffusion and MNB-dissolution sub-models, while accounting for the experimentally observed mobility and retention of nanobubbles in porous media (Borsky et al., 2024), would predict *p_O_2__(r, t)*, and hence the dynamic FAD boundary, at the temporal resolution of an irrigation cycle rather than at steady state. The predicted boundaries can finally be tested against root-density maps obtained from minirhizotrons, soil coring, or X-ray CT through the growing season, the framework predicting that roots proliferate preferentially within the FAD and that *η*_FAD_ scales with crop water productivity across contrasting soil textures and emitter layouts. Functional–structural plant models such as that of Renton et al. (2025) offer a natural platform for implementing cost–benefit decision rules and testing whether OFT-derived allocation reproduces observed architecture more faithfully than empirical parameterizations, though they yield falsifiable output only once supplied with the field-calibrated parameters above.

#### 4.2.3 Resolving the temporal dynamics of root-zone oxygen

A framework that treats root-zone oxygen as quasi-static cannot capture SDI, where each irrigation event transiently saturates the pore space and displaces air before drainage restores aeration, so that the amplitude, frequency, and duration of hypoxic pulses vary with scheduling, emitter discharge, hydraulic conductivity, and evaporative demand across the season. How the mechanism should be represented turns on three questions. The first is whether the architectural response integrates cumulative oxygen deficit, as in a degree-day model, or tracks instantaneous minima against a threshold; continuous planar-optode monitoring across irrigation cycles, with periodic root imaging, can distinguish them. The second is how developmental stage tunes sensitivity, since young elongating tips are likely more responsive to transient hypoxia than mature suberized segments, and factorial trials across vegetative, flowering, and fruiting stages would show whether the mechanism operates mainly during establishment or throughout the cycle. The third question is how warming may increase root and microbial respiratory demand (Ben-Noah and Friedman, 2018) and potentially affect MNB stability and pore connectivity, thereby altering the duration for which oxygen in the root zone remains above *p*_crit_. Quantifying the interaction between temperature and oxygen under climate projections developed for individual sites, using crop and soil water modeling approaches such as those applied by Wang et al. (2021), would help evaluate seasonal oxygen adequacy within the proposed FAD framework.

#### 4.2.4 From proof of concept to the field: economic and operational barriers

Two decades of controlled-environment evidence for positive agronomic responses have not translated into large-scale adoption of aerated SDI, and understanding why is a precondition for any credible case to expand it. The barriers are economic and operational, not agronomic. Venturi injectors, compressors, and MNB generators increase capital costs and require additional energy and maintenance. These costs have to be weighed against the agronomic benefits (Balea et al., 2026). Having both gas and liquid in the drip line alters emitter hydraulics; studies report conflicting effects on emitter clogging (Wang et al., 2025; Junejo et al., 2025). Field data on maintenance and life-cycle costs are still scarce (Balea et al., 2026). There is still no agreement on the best aeration rate, timing, or duration for particular crop-soil combinations, and most of the evidence still comes from greenhouse and pot experiments, not the multi-season, multi-site field trials needed to establish performance under commercial management. Taken together, these gaps point to a concrete transition path rather than an open-ended research agenda. If a standardized life-cycle cost analysis expressed the total cost of ownership of aerated relative to conventional SDI as a break-even yield increment (the minimum gain needed to recover the added investment), the meta-analytic yield responses already reported (Du et al., 2018; Wang et al., 2026b) could be judged against a clear economic benchmark. Coordinated validation networks, covering representative climates, soils, and cropping systems, could then check whether water-use efficiency, yield, root-zone dissolved oxygen, and clogging behave stably under commercial rather than researcher-optimized management. At the same time, engineering simplification (passive aeration, gravity-fed MNB generation, or intermittent schedules that keep oxygen above pcrit at minimum energy cost) could bring down the adoption threshold for resource-constrained systems. As things stand, adoption is constrained by the economics of field-scale aeration delivery, not by the underlying biology. The foraging-cost framework advanced here clarifies why oxygen should reshape root-zone performance; the remaining task is to establish, through the field-scale studies outlined above, whether this benefit can be realized at an acceptable cost under commercial management.
